# Potential mechanisms linking poverty alleviation and health: an analysis of benefit spending among recipients of the U.S. earned income tax credit

**DOI:** 10.1186/s12889-023-16296-1

**Published:** 2023-07-19

**Authors:** Rita Hamad, Joseph Yeb, Kaitlyn Jackson, Wendi Gosliner, Lia C.H. Fernald

**Affiliations:** 1 Department of Social & Behavioral Sciences, Harvard School of Public Health, 677 Huntington Avenue, 7th Floor, Boston, MA 02115 USA; 2 School of Public Health, Tufts University, Boston, MA USA; 3 Nutrition Policy Institute, Division of Agriculture and Natural Resources, University of California, Oakland, CA USA; 4 Division of Community Health Sciences, School of Public Health, University of California Berkeley, Berkeley, CA USA

**Keywords:** Social determinants of health, Poverty, Policy evaluation

## Abstract

**Background:**

The earned income tax credit (EITC) is the largest U.S. poverty alleviation program for low-income families, disbursed annually as a lump-sum tax refund. Despite its well-documented health impacts, the mechanisms through which the EITC affects health are not well understood. The objective of this analysis was to examine self-reported spending patterns of tax refunds among EITC recipients to clarify potential pathways through which income may affect health.

**Methods:**

We first examined spending patterns among 2020–2021 Assessing California Communities’ Experiences with Safety Net Supports (ACCESS) study participants (N = 241) and then stratified the analysis by key demographic subgroups.

**Results:**

More than half of EITC recipients reported spending their tax refunds on bills and debt (52.3%), followed by 49.4% on housing, and 37.8% on vehicles. Only 3.3% reported spending on healthcare. (Note: respondents could list more than one possible spending category.) Participants ages 30 + were more likely to spend on bills and debt relative to those ages 18–29 (57.6% versus 39.4%, respectively). Other subgroup analyses did not yield significant findings.

**Conclusions:**

Our findings suggest that EITC recipients primarily use their refunds on bills and debt, as well as on household and vehicle expenses. This supports the idea of the EITC as a safety net policy which addresses key social determinants of health.

**Supplementary Information:**

The online version contains supplementary material available at 10.1186/s12889-023-16296-1.

## Background

Poverty is a prevalent and persistent determinant of poor health [1]. The earned income tax credit (EITC) is the largest U.S. poverty alleviation program for families with children, disbursed in the form of a tax refund to working families with low income [2–4]. The EITC aims to reduce poverty while incentivizing employment. Younger, single, female heads of household with low educational attainment comprise the majority of EITC recipients [2, 5]. Disbursed as an annual lump-sum tax refund, the EITC amount for each family varies by earned income, marital status, and number of dependent children, with the mean federal refund at $2,411 in 2021 [5]. The EITC has positive effects on labor supply and income, and has also shown benefits for numerous health outcomes including infant birthweight, adult mental health, and food security [5–9].

The hypothesized pathways through which the EITC may affect health include the family investment mechanism (i.e., families have more money to spend on inputs like nutrition and healthcare) and the family stress model (i.e., depression and stress are lower because of greater household resources) [10]. Yet, there has been limited research on how EITC recipients actually spend their refunds [11, 12]. One prior study using the 1997–2006 waves of the Consumer Expenditure Survey found that households likely to be EITC recipients more often spent their money during tax season on durable goods—and particularly big-ticket items like household appliances—compared with other households [13]. Another survey of 200 EITC recipients in 2007 found over half of families intended to spend the refund on savings, although only 39% did so, while 84% paid down bills and debts [14]. To address this knowledge gap, we conducted a cross-sectional survey of a diverse sample of EITC-eligible California families to understand spending patterns and enhance understanding of this critical policy’s health effects.

## Methods

### Data collection

Data were drawn from the Assessing California Communities’ Experiences with Safety Net Supports (ACCESS) Study, which involved survey-based interviews with EITC-eligible California families with children during August 2020-May 2021 (N = 241). Study procedures for the ACCESS Study have been described previously [15]. For the purposes of this sub-study, the sample was restricted to individuals who had their 2019 tax returns available for review, to confirm receipt of the EITC. All study protocols were approved by the California Committee for the Protection of Human Subjects and the University of California, Berkeley institutional review board. Informed consent was obtained from all subjects.

### Survey implementation

The survey included questions on sociodemographic characteristics and data from tax forms. Participants self-identified their race/ethnicity, and this was categorized into Hispanic/Latinx, non-Hispanic Black, non-Hispanic White, and non-Hispanic other. EITC recipients were asked how they spent their 2019 tax refunds, which they would have received in early 2020. This was included as an open-ended question, and interviewers marked responses mentioned by recipients from the following list of 12 spending categories: housing (e.g., mortgage, rent), utilities, home-related repairs or improvements (e.g., construction, appliances), clothing, vehicle-related expenses, healthcare and health insurance payments, non-health insurance policies, educational expenses, entertainment expenses, food and beverages, savings and investments, or other (for which they would specify the expense). All responses designated as “other” were reviewed, cleaned, and further categorized by study staff, yielding three additional categories: bills, debt, and children’s needs. Respondents could list more than one possible spending category, and they might select two categories to represent a single expense (e.g., debt and education for an educational loan).

Finally, 11 categories were created by combining several categories noted above that addressed related concepts and collapsing them to: housing, vehicles, bills and debt, retail consumption, food, savings/investments, children’s needs, home repairs/improvements, education, healthcare and health insurance, and miscellaneous (Supplemental Table 1).

### Data analysis

First, we calculated descriptive statistics. Then, we tabulated the percentage of participants who reported spending their refund on the categories above.

We also hypothesized that the EITC may be more valuable for individuals with fewer resources due to structural racism and discrimination, so we then stratified analyses by sociodemographic characteristics: age, gender, race/ethnicity, marital status, employment status, educational attainment, and refund size. Income and refund size were dichotomized by splitting them at the mean. We conducted two-tailed tests of proportions to assess whether spending was statistically significantly different across subgroups. Differences in spending patterns may inform our understanding of why larger health benefits have been observed for some groups, like Black women or those with lower educational attainment [16–18].

## Results

### Sample characteristics

Of the 241 participants, most (93.8%) were female (Table 1). Approximately half were Latinx/Hispanic, and one quarter Black. One-fifth had a bachelor’s degree or higher. Median annual adjusted gross income was $19,257 (IQR 11,718 − 30,000). One quarter of respondents reported being primarily unemployed in 2019; however, EITC income-specific eligibility was assessed at the household level (i.e., respondent + spouse), rather than at the individual level, so their household still earned enough income to qualify for the EITC. The median 2019 EITC amount was $3,194 (IQR 1,712-4,789). The mean 2019 EITC amounts nationally and in California were $2,461 and $2,297, respectively, although these included individuals without children, who receive smaller EITC benefits [2].

**Table 1:** Participant Characteristics

Variables	Percent or Median (IQR)
Female	93.8
Race/ethnicity	
Latinx/Hispanic	50.6
Non-Hispanic Black	23.7
Non-Hispanic White	13.3
Non-Hispanic Other	12.4
Age (years)	
18–29	29.5
30+	70.5
Foreign-born	24.1
Primary language English	84.0
Num. adults in household	2 (IQR 1–2)
Num. children in household	2 (IQR 1–3)
Adjusted gross income in 2019 ($US)	19257.0 (IQR 11,718–30,000)
College education or more	19.9
Work status	
Full-time	43.3
Part-time	31.7
Not working	25.0
Married	52.7
Earned income tax credit amount in 2019 ($US)	3194.0 (IQR 1712.0-4789.0)

N = 241. Sample drawn from the Assessing California Communities’ Experiences with Safety Net Supports (ACCESS) Study, which interviewed economically disadvantaged California families with young children and verified receipt of the earned income tax credit via tax returns.

### Refund spending

About half of respondents spent their refunds on bills and debt (52.3%) and housing (49.4%), followed by 37.8% on vehicles (Table 2). Savings and investments (10.4%), education (5.4%), and healthcare and health insurance (3.3%) were the least common. Respondents could list more than one possible spending category, so the total in this table sums to greater than 100%. In stratified analyses (Supplemental Table 2), there were generally no differences across spending categories based on participant characteristics; we summarize the few exceptions here. In subgroup analyses by race/ethnicity, participants of other races were more likely to spend their refund on education (16.7%, p = 0.03) and food/beverages (40.0%; p = 0.02) compared with Latinx, Black, and White participants. In subgroup analyses by age, study participants ages 30 or older were more likely to spend tax refunds on bills and debt compared with participants aged 18–29 (57.6% versus 39.4, p = 0.01). In subgroup analyses by employment status, employed participants were more likely to spend their refund on miscellaneous items (6.1%, p = 0.02) compared with unemployed participants. There were no differences in refund spending by income or EITC refund size.

**Table 2:** Tax Refund Spending among EITC Recipients, by Spending Category

Spending Categories	N (%)
Bills and debt	126 (52.3)
Housing	119 (49.4)
Vehicles	91 (37.8)
Retail consumption	62 (25.7)
Food	57 (23.7)
Children’s needs	29 (12.0)
Home repairs and improvements	29 (12.0)
Savings and investments	25 (10.4)
Education	13 (5.4)
Healthcare and health insurance	11 (4.6)
Miscellaneous	8 (3.3)

N = 241. Sample drawn from the Assessing California Communities’ Experiences with Safety Net Supports (ACCESS) Study, which interviewed economically disadvantaged California families with young children and verified receipt of the earned income tax credit via tax returns. Recipients could report spending on more than one category. Details on spending categories are included in the Supplement.

## Discussion

This study interviewed Californians with low income who were recipients of the EITC—the largest U.S. poverty alleviation program for families with children—to understand how they spent their tax refunds. Participants reported spending on basic needs like housing, vehicles, bills, and debt. California has a higher-than-average cost-of-living, with the median cost of housing at nearly twice the national average,[19] which likely explains housing as the second-largest spending category. Prior studies using administrative data and older surveys corroborate these findings, where recipients of the EITC and other income support programs allocated benefits to durable goods like cars or immediate concerns like bills [5, 13, 14]. Participants reported minimal spending on healthcare, similar to prior work finding no short-run changes in healthcare utilization after EITC receipt [20].

Few statistically significant subgroup differences emerged, perhaps due to the small sample size, or because spending was similar across subgroups. These few exceptions should be interpreted with caution in light of multiple hypothesis testing.

This study’s primary strength is the first-hand assessment of tax refund spending from actual EITC recipients as verified by review of tax returns. Limitations include recruitment through convenience sampling, so results may not be representative or generalizable. Additionally, data collection occurred during the COVID-19 pandemic; given the circumstances, spending may differ from pre-pandemic or post-pandemic periods (e.g., due to the receipt of stimulus checks), although this study provides insight into spending among vulnerable families during this critical time. Additionally, we did not ask for spending amounts in each category, nor itemized spending within categories, such as the exact type of debt being paid. This could potentially mask EITC spending on medical debt, a type of debt that makes up a large portion of debt collections and that increases in months with higher income [21, 22]. For this reason, we are unable to assess the connection between the EITC and health through medical debt. Finally, self-reported information may be subject to standard reporting biases, like social desirability.

## Conclusion

Previous studies have found that the EITC improves health. By examining how EITC recipients spend their tax refunds and finding that the majority of spending on bills and debt, housing and vehicles, this study contributes evidence to understand the possible mechanisms for this association. Future research using larger samples could build on our exploratory subgroup analyses to examine whether individual- and area-level characteristics affect spending patterns.

## Electronic supplementary material

Below is the link to the electronic supplementary material.

Supplementary Material 1

## Data Availability

The datasets used and/or analyzed during the current study are available from the corresponding author on reasonable request.

## Abbreviations

ACCESS: Assessing California Communities’ Experiences with Safety Net Supports
EITC: Earned Income Tax Credit
