# Supplementary material for: Potential mechanisms linking poverty alleviation and health: an analysis of benefit spending among recipients of the U.S. earned income tax credit
Source: BMC Public Health. 2023 Jul 19;23:1385. doi: 10.1186/s12889-023-16296-1 (PMC10354901; doi:10.1186/s12889-023-16296-1)
Supplement: Supplementary file 1 — Supplementary Material 1 [file 12889_2023_16296_MOESM1_ESM.pdf]

# SUPPLEMENT

## Supplemental Methods

*Data Collection*

Briefly, participants were recruited in partnership with community-based organizations and completed an online eligibility screening questionnaire. Those deemed EITC-eligible and who met other inclusion criteria (e.g., California residence) were interviewed in English or Spanish by trained study staff (N=499). Interviewers also reviewed 2019 tax returns for participants who had filed taxes to verify EITC receipt. This analysis was restricted to participants with verified EITC receipt (N=241). The screening questionnaire and survey were conducted and stored in Qualtrics.

Participants self-identified their race/ethnicity, and this was categorized into Hispanic/Latinx, non-Hispanic Black, non-Hispanic White, and non-Hispanic other. The latter category includes those of other or multiple races; more granular categories could not be included due to small cell sizes.

*Data Analysis*

Data analysis was conducted during 2021-2022.

## Supplemental Tables

**Supplemental Table 1. Collapsing of Survey-Derived Spending Categories into Analytic Categories**

| *Original Survey-Derived Spending Categories* | *Revised Analytic Spending Categories* |
|---|---|
| Vehicles | Vehicles |
| Utilities, Insurance/Bills, Debt | Bills and debt |
| Clothing, Entertainment | Retail consumption |
| Housing | Housing |
| Food | Food |
| Savings and investments | Savings and investments |
| Children's needs | Children's needs |
| Home repairs/improvements | Home repairs/improvements |
| Education | Education |
| Healthcare and health insurance | Healthcare and health insurance |
| Miscellaneous | Miscellaneous |

N = 241. Sample drawn from the Assessing California Communities' Experiences with Safety Net Supports (ACCESS) Study, which interviewed economically disadvantaged California families with young children and verified receipt of the earned income tax credit via tax returns.

**Supplemental Table 2. Tax Refund Spending by Spending Category Among EITC Recipients, Stratified by Sociodemographic Characteristics**

| | *Spending Categories, N (%)* | | | | | | | | | | |
|---|---|---|---|---|---|---|---|---|---|---|---|
| *Subgroups* | Vehicles | Housing | Bills & debt | Retail consumption | Food | Savings & investments | Children's needs | Home repairs | Education | Healthcare & health insurance | Misc. |
| Race/Ethnicity | | | | | | | | | | | |
| Latinx/Hispanic | 51 (41.8) | 57 (46.7) | 60 (49.2) | 32 (26.2) | 26 (21.3) | 12 (9.8) | 12 (9.8) | 12 (9.8) | 4 (3.3) | 6 (4.9) | 2 (1.6) |
| Black | 20 (35.1) | 30 (52.6) | 32 (56.1) | 16 (28.1) | 10 (17.5) | 6 (10.5) | 10 (17.5) | 9 (15.8) | 4 (7.0) | 2 (3.5) | 4 (7.0) |
| White | 11 (34.4) | 20 (62.5) | 16 (50.0) | 7 (21.9) | 9 (28.1) | 4 (12.5) | 5 (15.6) | 5 (15.6) | 0 (0.00) | 2 (6.3) | 0 (0.0) |
| Other | 9 (30.0) | 12 (40.0) | 18 (60.0) | 7 (23.3) | 12 (40.0)* | 3 (10.0) | 2 (6.7) | 3 (10.0) | 5 (16.7)** | 1 (3.3) | 2 (6.7) |
| Marital Status | | | | | | | | | | | |
| In relationship | 67 (35.3) | 33 (48.5) | 35 (51.5) | 16 (23.5) | 19 (27.9) | 7 (10.3) | 6 (8.8) | 6 (8.8) | 6 (8.8) | 3 (4.4) | 3 (4.4) |
| Single | 24 (38.7) | 86 (49.7) | 91 (52.6) | 46 (26.6) | 38 (22.0) | 18 (10.4) | 23 (13.3) | 23 (13.3) | 7 (4.1) | 8 (4.6) | 5 (2.9) |
| Educational Attainment | | | | | | | | | | | |
| High school or less | 23 (35.4) | 34 (52.3) | 29 (44.6) | 19 (29.2) | 15 (23.1) | 6 (9.2) | 7 (10.8) | 6 (9.23) | 3 (4.6) | 3 (4.6) | 3 (4.6) |
| Some college | 48 (37.5) | 57 (44.5) | 70 (54.7) | 35 (27.3) | 28 (21.9) | 13 (10.2) | 19 (14.8) | 18 (14.1) | 7 (5.5) | 6 (4.7) | 5 (3.9) |
| College or more | 20 (41.7) | 28 (58.3) | 27 (56.2) | 8 (16.7) | 14 (29.2) | 6 (12.5) | 3 (6.3) | 5 (10.4) | 3 (6.3) | 2 (4.2) | 0 (0.0) |
| Age | | | | | | | | | | | |
| 18-29 | 33 (46.5) | 31 (43.7) | 28 (39.4) | 20 (28.2) | 16 (22.5) | 7 (9.9) | 5 (7.0) | 12 (16.9) | 0 (0.0) | 2 (2.8) | 3 (4.2) |
| 30+ | 58 (34.1) | 88 (51.8) | 98 (57.6)** | 42 (24.7) | 41 (24.1) | 18 (10.6) | 24 (14.1) | 17 (10.0) | 13 (7.7)* | 9 (5.3) | 5 (2.9) |
| Work Status | | | | | | | | | | | |
| Employed | 43 (38.1) | 59 (46.8) | 55 (55.6) | 29 (26.2) | 28 (23.0) | 12 (10.3) | 14 (12.3) | 17 (9.52) | 5 (4.4) | 4 (5.6) | 7 (0.8) |
| Unemployed | 48 (37.3) | 59 (51.8) | 70 (48.2) | 33 (25.4) | 29 (24.6) | 18 (10.5) | 15 (11.9) | 12 (14.9) | 8 (6.4) | 7 (3.5) | 1 (6.1)* |
| EITC Amount | | | | | | | | | | | |
| < Mean | 46 (39.0) | 54 (45.8) | 61 (50.8) | 32 (26.3) | 30 (22.0) | 13 (11.0) | 17 (14.4) | 13 (45.8) | 7 (5.9) | 6 (3.4) | 4 (3.4) |
| ≥ Mean | 44 (36.4) | 64 (52.9) | 65 (52.9) | 30 (25.6) | 27 (25.6) | 12 (9.1) | 12 (9.9) | 16 (52.9) | 6 (5.0) | 5 (5.0) | 4 (3.3) |
| Annual Household Income | | | | | | | | | | | |
| < Mean | 47 (37.6) | 63 (50.4) | 61 (48.8) | 32 (25.6) | 26 (24.0) | 13 (10.4) | 15 (12.0) | 18 (14.4) | 6 (4.8) | 6 (4.8) | 3 (2.4) |
| ≥ Mean | 44 (37.9) | 56 (48.3) | 65 (56.0) | 30 (25.9) | 31 (23.3) | 11 (10.3) | 14 (12.1) | 11 (9.5) | 7 (6.0) | 5 (4.3) | 5 (4.3) |

**Note:** **p < 0.01, *p<0.05 compared with all other groups.
N = 241. Sample drawn from the Assessing California Communities' Experiences with Safety Net Supports (ACCESS) Study, which interviewed economically disadvantaged California families with young children and verified receipt of the EITC via tax returns. Subgroup analyses involve two-tailed t-tests. EITC: earned income tax credit.
